# Extracellular Vesicle Proteomes Shed Light on the Evolutionary, Interactive, and Functional Divergence of Their Biogenesis Mechanisms

**DOI:** 10.3389/fcell.2021.734950

**Published:** 2021-10-01

**Authors:** Hyobin Julianne Lim, Haejin Yoon, Hyeyeon Kim, Yun-Won Kang, Ji-Eun Kim, Oh Youn Kim, Eun-Young Lee, Jean-Claude Twizere, Janusz Rak, Dae-Kyum Kim

**Affiliations:** ^1^Center for Personalized Medicine, Roswell Park Comprehensive Cancer Center, Buffalo, NY, United States; ^2^Department of Cell Biology, Blavatnik Institute and Harvard Medical School, Boston, MA, United States; ^3^Princess Margaret Cancer Centre, University Health Network, Toronto, ON, Canada; ^4^Program in Developmental and Stem Cell Biology, The Hospital for Sick Children, Toronto, ON, Canada; ^5^Department of Molecular Genetics, University of Toronto, Toronto, ON, Canada; ^6^College of Medicine, Yonsei University, Seoul, South Korea; ^7^Infection and Immunity Research Laboratory, Metabolic Regulation Research Center, Korea Research Institute of Bioscience and Biotechnology, Daejeon, South Korea; ^8^Laboratory of Viral Interactomes, GIGA Institute, University of Liège, Liege, Belgium; ^9^TERRA Teaching and Research Centre, University of Liège, Liege, Belgium; ^10^Research Institute of the McGill University Health Centre, Glen Site, McGill University, Montreal, QC, Canada

**Keywords:** extracellular vesicle, biogenesis, purification, evolution, network

## Abstract

Extracellular vesicles (EVs) are membranous structures containing bioactive molecules, secreted by most cells into the extracellular environment. EVs are classified by their biogenesis mechanisms into two major subtypes: ectosomes (enriched in large EVs; lEVs), budding directly from the plasma membrane, which is common in both prokaryotes and eukaryotes, and exosomes (enriched in small EVs; sEVs) generated through the multivesicular bodies via the endomembrane system, which is unique to eukaryotes. Even though recent proteomic analyses have identified key proteins associated with EV subtypes, there has been no systematic analysis, thus far, to support the general validity and utility of current EV subtype separation methods, still largely dependent on physical properties, such as vesicular size and sedimentation. Here, we classified human EV proteomic datasets into two main categories based on distinct centrifugation protocols commonly used for isolating sEV or lEV fractions. We found characteristic, evolutionarily conserved profiles of sEV and lEV proteins linked to their respective biogenetic origins. This may suggest that the evolutionary trajectory of vesicular proteins may result in a membership bias toward specific EV subtypes. Protein–protein interaction (PPI) network analysis showed that vesicular proteins formed distinct clusters with proteins in the same EV fraction, providing evidence for the existence of EV subtype-specific protein recruiters. Moreover, we identified functional modules enriched in each fraction, including multivesicular body sorting for sEV, and mitochondria cellular respiration for lEV proteins. Our analysis successfully captured novel features of EVs embedded in heterogeneous proteomics studies and suggests specific protein markers and signatures to be used as quality controllers in the isolation procedure for subtype-enriched EV fractions.

## Introduction

Extracellular vesicles (EVs) are membrane-bound particles that are secreted by cells across the evolutionary spectrum (Gill et al., 2019). EVs enable the export of proteins, RNAs, lipids, and other biomolecules (Oliveira et al., 2010), protected from degradation by proteases and RNases in the extracellular fluid (O’Brien et al., 2020). EVs mediate communications systemically between organs or locally between cells (Al-Nedawi et al., 2008). However, EVs are immensely heterogeneous and our understanding of how their different subtypes are formed and how specific cargoes are selected for transport and delivered remains limited. Thus, it is important to differentiate the heterogeneous populations of EVs to understand the physiology of vesicles and their potential use, including specific biomarkers in disease.

There are two major subtypes of EVs based on their biogenesis and size (Cocucci and Meldolesi, 2015): (i) Ectosomes (100–1000 nm in diameter) bud from the cellular surface and are frequently referred to as microvesicles in eukaryotes, or as outer membrane vesicles in prokaryotes and (ii) exosomes (30–100 nm) arise within endosome-related intraluminal vesicles, which are then released from cells upon recruitment of multivesicular bodies to the inner layer of the plasma membrane (Colombo et al., 2014; van Niel et al., 2018). Both prokaryotic and eukaryotic cells secrete EVs, even though the biogenesis of exosomes is thought to be specific to eukaryotic cells due to the requirement for endosomal compartment (Deatherage and Cookson, 2012). However, the impact of evolutionary characteristics of biogenesis mechanisms on the vesicular cargo composition has not been explored.

To uncover mechanisms of EV biogenesis, a myriad of EV proteomic studies have been conducted under various conditions, from different cellular sources, and on different subtypes of EVs. EV subtypes may have different physiological roles and compositions (Kanada et al., 2015; Kowal et al., 2016), but it is difficult to dissect their differences on an empirical basis. Indeed, current isolation methods are limited in separating EV subtypes exclusively (Li et al., 2019), and the true predicted heterogeneity of EVs probably exceeds the traditional classification by orders of magnitude (Choi et al., 2019). Differential ultracentrifugation (DUC) is most commonly used to pre-clear debris and unwanted EV subtypes and/or concentrate the target EV subtype (Choi et al., 2013, 2015; Meldolesi, 2018). Although it is recommended to combine DUC methods with other purification methods, such as size-exclusion chromatography and affinity purification (Jeppesen et al., 2019), the choices of molecular markers used to pull down the desired EV subpopulations are often arbitrary and result in biased sampling that might introduce experimental artifacts and skew EV profiles. This emerging challenge may be addressed by integrating multiple datasets from various sources to validate common isolation methods and markers.

We hypothesize that the proteomic composition of ectosomes and exosomes fundamentally differs in an evolutionary, functional, and network-topological manner, due to their distinct core biogenesis mechanisms. We classified EV proteomic datasets available in the EVpedia database (Kim et al., 2013, 2015a,2015b) into two datasets – a smaller exosome-enriched fraction (small EV; sEV) and a larger ectosome-enriched fraction (large EV; lEV) – using the frequently used DUC cutoffs for analyzing specific EV subtypes. We validated our approach by comparing the identification frequency of popular exosomal and ectosomal markers in our classified sEV and lEV datasets. Moreover, our findings demonstrate that sEV and lEV datasets exhibit significant differences in: (i) degree of evolutionary conservation of proteins, (ii) topology of protein–protein interactions (PPIs), and (iii) enrichment in biological functions. While EVs are more readily secreted by cancer cells and represent a promising diagnostic analyte (Kosaka et al., 2019; Hoshino et al., 2020; Kalluri and LeBleu, 2020), differential expression of biomarkers between a diseased and normal cell may arise from cross-contamination between EV subtypes. Therefore, an integrated computational approach for analyzing their larger protein networks not only validates isolation methods but may also reveal a more comprehensive understanding of EV subtypes beyond the detection of individual protein markers, enabling discovery of novel cancer signatures and potential therapeutic targets.

## Materials and Methods

### Classification of Extracellular Vesicle Proteomic Datasets Into Small Extracellular Vesicle and Large Extracellular Vesicle Datasets Based on Differential Ultracentrifugation Methods

We constructed an integrated EV proteomic dataset by compiling the entire list of shotgun proteomic datasets (Supplementary Table 1A) from EVpedia (Kim et al., 2013, 2015a,2015b), updated on April 30, 2018. We classified 485 human datasets based on DUC conditions used for purifying the EV samples. Given several methods to enrich lEVs at 10,000 × *g* (Kowal et al., 2016), we assumed that if the EV fraction has undergone pre-clearing of 10,000 × *g* or more, the sample has been cleared of most lEVs. We set the ultracentrifugation speed cut-off under 20,000 × *g* for sedimenting lEVs and over 100,000 × *g* for sEVs. Standards for classification can be found in detail in Supplementary Table 1B, and methods that did not meet these standards were excluded (Supplementary Table 1C). Common Tree tool from NCBI taxonomy database (latest updated on August 22, 2021) (Schoch et al., 2020) was used to generate a phylogenetic tree of species included in the EVpedia database (Kim et al., 2013, 2015a,2015b) and further visualized with the iTOL tool (version 5) (Letunic and Bork, 2021).

### Analysis of Exosomal and Ectosomal Markers in Large Extracellular Vesicle and Small Extracellular Vesicle Proteomes

We identified 10 conventional molecular markers each for human ectosomes and exosomes from an extensive literature search (Muralidharan-Chari et al., 2009; Lee et al., 2012; Surman et al., 2017; Meldolesi, 2018; Jeppesen et al., 2019; Rogers et al., 2020). We defined “Identification frequency in sEV datasets (fsEV)” and “Identification frequency in lEV datasets (flEV)” of every vesicular protein in the integrated dataset, which is the number of times that the protein was identified in a particular subset, divided by the total number of sEV or lEV studies in the subset, respectively (Supplementary Table 2). We used Fisher’s exact test in MatLab (version 2016a) to compare individual markers between the datasets and Mann–Whitney *U* test to compare the identification of all the markers via GraphPad Prism (version 8.2). A scatter plot of fsEV and flEV was visualized in MatLab.

### Evaluation of Evolutionary Conservation of Small Extracellular Vesicle and Large Extracellular Vesicle Protein Components

To evaluate the evolutionary conservation of proteins enriched in sEV and lEV, we used identification counts (ICs) of orthologous proteins in prokaryotes and eukaryotes across the entire dataset from EVpedia (Supplementary Table 2). IC_eu_ (identification count in eukaryotic EV datasets) and IC_pro_ (identification count in prokaryotic EV datasets) for proteins that showed higher identification frequency in each subset [log2(fsEV/flEV) > 0.1 for sEV; log2(fsEV/flEV) < -0.1 for lEV] were used for analysis. Statistical analysis was performed using Mann–Whitney *U* test in GraphPad Prism (version 8.2).

### Protein–Protein Interaction Network Analysis of Vesicular Proteins

To construct a unified protein–protein interactome network, we integrated Affinity Purification-Mass Spectrometry (AP-MS)-based network from the BioPlex project (version 3.0) based on two distinct cell lines of HEK293T and HCT112 (Huttlin et al., 2015, 2017, 2021) and Yeast Two-Hybrid (Y2H)-based network from Human Reference Interactome (HuRI) project (Rual et al., 2005; Rolland et al., 2014; Luck et al., 2020; Supplementary Table 3). The Spearman correlation of fsEV/flEV ratio between bait and prey proteins of interacting pairs and their *P*-value were calculated by MatLab (version 2016a). The network was visualized by Cytoscape (version 3.8.2) (Shannon et al., 2003).

### Functional Enrichment by Gene Set Enrichment Analysis

We constructed two separate gene lists ranked by (i) relative identification in sEV and lEV datasets [log2(fsEV/flEV)] or (ii) relative identification counts in human vesicular proteomes (IC_human_ - an average of IC_human_) for proteins that were equally abundant [| log2(fsEV/flEV)| < 0.1] in sEV and lEV datasets. Biological functions enriched in each classification category were determined using Gene Set Enrichment Analysis (GSEA; version 4.1.0) (Subramanian et al., 2005). Functional annotations that were significantly enriched (false discovery rate < 0.05) were visualized using Cytoscape (version 3.8.2) (Shannon et al., 2003) apps, EnrichmentMap (version 3.3.2) (Merico et al., 2010), and AutoAnnotate (version 1.3.3) (Kucera et al., 2016).

## Results

### Small Extracellular Vesicle and Large Extracellular Vesicle Datasets Exhibit a Differential Separation of Exosomal and Ectosomal Markers

**FIGURE 1:**
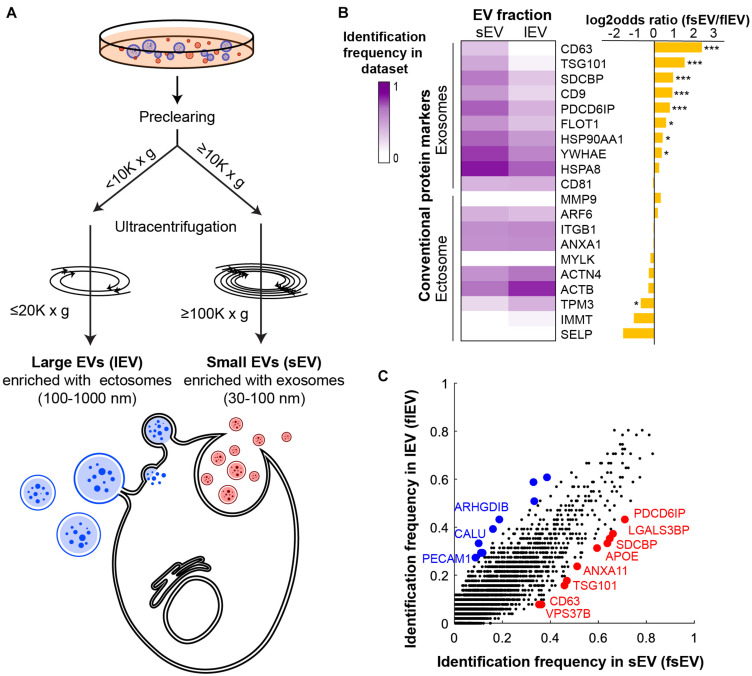
Protein markers of ectosomes and exosomes show different identification frequencies in human small extracellular vesicle (sEV) and large EV (lEV) proteomic datasets. **(A)** Schematic of DUC criteria shows how we classified human proteomic datasets into sEV and lEV datasets. **(B)** Identification frequency of literature-surveyed EV protein markers in sEV (fsEV) and lEV (flEV) datasets supports the validity of our classification methods. *P < 0.05; ***P < 0.001. **(C)** A scatter plot comparing fsEV and flEV of all human vesicular proteins deposited in EVpedia shows that known exosomal and ectosomal markers correctly identify EV subtype enriched in each fraction. Protein markers with the 10 highest differences between their identification frequencies in sEV and lEV are highlighted by red and blue for fsEV and flEV, respectively.

DUC is a widely used purification method to separate exosome- or ectosome-enriched EV fractions (Kowal et al., 2016). Ectosome-enriched lEV fraction is often sedimented under 10,000 × *g* centrifugal force and exosome-enriched sEVs require over 100,000 × *g* preceded by pre-clearance of lEVs (Greening et al., 2015; Jeppesen et al., 2019). To maximize the size of input data for proper statistical testing, we set slightly more permissive standards of ≥100,000 × *g* with ≥10,000 × *g* of pre-clearance for sEV datasets and ≤20,000 × *g* and <10,000 × *g* pre-clearance for lEV datasets (Figure 1A and Supplementary Tables 1B,C). We performed our analyses on human datasets to account for variability of size and density between EVs of different species. A total of 485 human proteomic datasets have been classified into 51 lEV and 203 sEV datasets (Supplementary Table 1A). We then defined “Identification frequency in sEV datasets (fsEV)” and “Identification frequency in lEV datasets (flEV)” of every vesicular protein identified in the integrated 254 human datasets in which the number of datasets that identified the protein divided by the whole number of sEV or lEV datasets, respectively. These measures might provide us with a systematic evaluation of each protein’s tendency to be present in sEVs or lEVs.

With an intent to validate our classification strategy while also evaluating the robustness of the common markers used, we examined enrichments of previously known exosomal and ectosomal markers in each fraction (Muralidharan-Chari et al., 2009; Lee et al., 2012; Surman et al., 2017; Meldolesi, 2018; Jeppesen et al., 2019; Kalluri and LeBleu, 2020; Rogers et al., 2020). Exosomal markers are generally restricted to the endocytic pathway. However, ectosomal markers are more diverse and the enriched proteins depend heavily on the samples and cell types from which they have originated (Lee et al., 2012; Liu and Williams, 2012; Sluijter et al., 2014). Markers representing exosomes included tetraspanins (CD63, CD81, and CD9), endomembrane network-associated proteins (FLOT1, PDCD6IP, SDCBP, TSG101, and YWHAE), and heat shock proteins (HSPA8 and HSP90AA1). Although molecular markers that distinguish ectosomes are less well defined, we selected actins (ACTB and ACTN4), myosins (MYLK and TPM3), selectin (SELP), lipid membrane remodeling molecule (ARF6), protease (MMP9), integrin (ITGB1), mitochondrial protein (IMMT), and annexin (ANXA1) as relatively ectosome-specific markers. Overall, we saw significant differences in the expression of markers between sEV and lEV fractions (Figure 1B). We showed that the most common exosome-specific markers were more readily identified in sEV datasets (Figure 1B and Supplementary Figure 1A). Some ectosomal markers such as TPM3 showed higher identification in lEV datasets (Figure 1B), but the overall identification frequency of 10 ectosomal markers was not significantly enriched in lEV compared to sEV datasets (Supplementary Figure 1B). Surprisingly, CD81 and ARF6, which are among the most common markers for exosomes and ectosomes, respectively, did not show a statistical difference between the datasets. While CD63 and TPM3 show high exclusivity in their respective datasets, they have a low frequency of identification. Therefore, we propose that multiple markers should be considered together when isolating EVs using affinity purification, as there are no good stand-alone markers that ensure high identification and specificity across sample sets.

We next compared fsEV and flEV for all the vesicular proteins deposited in EVpedia (Figure 1C). Among the top 10 proteins enriched in lEVs (colored blue in Figure 1C; *P* < 0.001 by Fisher’s exact test), only two proteins were previously known as ectosomal markers: ARHGDIB (Cocucci and Meldolesi, 2011; Surman et al., 2017; Meldolesi, 2018) and PECAM1 (Kalra et al., 2016; Meldolesi, 2018; Słomka et al., 2018). CALU is a calcium-binding protein, which has been shown to regulate ectosomal budding at the plasma membrane (Surman et al., 2017). All seven remaining proteins are mitochondrial proteins (ATP5F1B, HSPD1, ATP5F1A, GOT2, ATP5PO, DLD, and UQCRC2), previously reported to be loaded into ectosomes (Mittelbrunn and Sánchez-Madrid, 2012; Phinney et al., 2015; Torralba et al., 2016). Among the top 10 enriched proteins in exosomes (colored red in Figure 1C), we found five endosomal pathway regulators (PDCD6IP, SDCBP, ANXA11, TSG101, and VPS37B) which are pivotal for exosome biogenesis, and CD63, a widely known exosomal tetraspanin marker. LGALS3BP has been found in diverse cancer-derived exosomes (Escrevente et al., 2013; Ung et al., 2014; Dutta et al., 2015; Gomes et al., 2015; Song et al., 2021), and APOE has been found in exosomes released from macrophages (Zheng et al., 2018) and neurons (Nikitidou et al., 2017; Mathews and Levy, 2019; Peng et al., 2019). This comparative analysis shows that our classification pipeline can successfully categorize proteomic datasets and suggests that systematic analysis of published proteomic studies is a valuable approach for discovering novel markers for ectosome and exosomes.

### Proteins Enriched in Large Extracellular Vesicles and Small Extracellular Vesicles Have Differential Evolutionary Conservation Profiles

**FIGURE 2:**
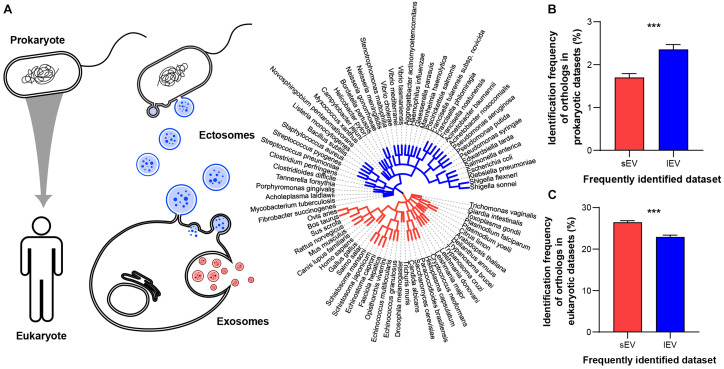
Protein components of sEVs and lEVs show different evolutionary conservation. **(A)** Schematic diagram of EV evolutionary conservation (left two panels). EVs derived from different biogenesis mechanisms show different availability in two different kingdoms. Phylogenetic tree (right panel) illustrates the evolutionary trajectory of species included in the EVpedia database. The topology is based on EVpedia (Kim et al., 2013, 2015a,2015b). Branch lengths are proportional to the number of non-synonymous substitutions per site. Red and blue branches represent eukaryotes and prokaryotes, respectively. **(B)** Proteins that are more frequently identified in lEV fractions are more conserved in the prokaryotic (IC_pro_/141 prokaryotic datasets) kingdom, **(C)** while proteins that are more frequently identified in sEV fraction are more conserved within the eukaryotic (IC_eu_/656 eukaryotic datasets) kingdom. ***P < 0.001, *n* = 3361 and 3028 proteins specifically enriched in small and large EV fractions, respectively.

Exosome forms in the late endosome, an organelle unique to eukaryotes, and transmits small intraluminal vesicle contents to the lysosome or the plasma membrane (Harris, 1986; Edgar, 2016). In contrast, ectosomes bud directly from the plasma membrane (Cocucci and Meldolesi, 2011), a mechanism used by both eukaryotes and prokaryotes (Deatherage and Cookson, 2012). Therefore, we hypothesized that the biogenesis mechanisms of EV subtypes correlate with kingdom-specific evolutionary conservation of proteins that are secreted via EVs (Figure 2A). To define a measure for the kingdom-specific evolutionary conservation, we used the identification count provided by EVpedia of proteins that had a higher tendency to be in sEV or lEV fraction. For example, “identification count in prokaryotes (IC_pro_)” indicates the number of prokaryotic EV proteomic datasets that had identified a particular protein’s orthologs, as defined by the EggNOG database (Jensen et al., 2008; Supplementary Table 2). A higher identification count indicates that the EV protein is more conserved within the EVs of eukaryotes or prokaryotes. The phylogenetic tree shows how species that have EV proteomes available in EVpedia are evolutionarily related (Figure 2A, right). In general, identification counts for prokaryotic proteins are lower, meaning that most human EV proteins were not well conserved in prokaryotes. As we predicted, ectosome-enriched lEV proteins show higher conservation in prokaryotes (Figure 2B) and lower conservation in eukaryotes (Figure 2C). Conversely, exosome-enriched sEV proteins are conserved more in eukaryotes and less in prokaryotes (Figures 2B,C). Despite being significantly different, the differences may be small due to EV proteins that do not participate in biogenesis mechanisms being conserved in both sEV and lEV fractions. Nevertheless, our result demonstrates that the current purification methods for EV subtypes provide us with the evolutionarily distinct EV proteomes and further suggests that evolutionary trajectories of vesicular proteins may be an important determinant influencing cargo selection.

### Network Analyses Reveal That Protein–Protein Interaction Networks of Small Extracellular Vesicles and Large Extracellular Vesicles Proteins Have High Intra-Fractional and Low Inter-Fractional Enrichment of Interactions

**FIGURE 3:**
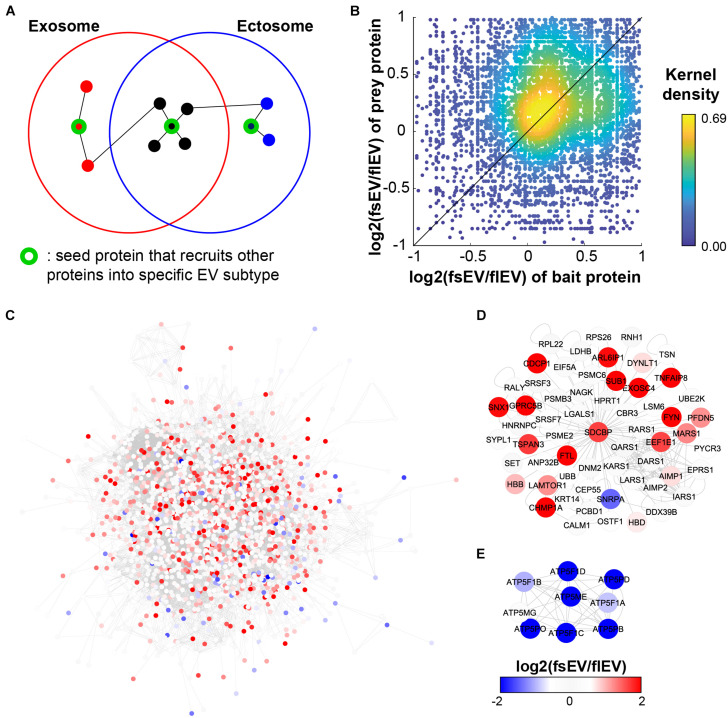
Network analysis shows enrichment of protein–protein interactions (PPIs) among the same EV fractions. **(A)** The schematic figure shows how EV fraction-specific “seed proteins” affect the PPI enrichment among the components of each EV fraction. **(B)** PPIs are enriched among the components in the same EV fraction, not between the different fractions; Spearman correlation ρ = 0.1319; *P* < 10^– 42^. **(C)** Integrated PPI network shows an exclusive cluster of EV proteins enriched in sEVs and lEVs. We have two examples of “seed protein” candidates: SDCBP for sEVs **(D)** and ATP5ME for lEVs **(E)**.

A biased PPI network resulting from the over-representation of popular genes may limit our ability to explore new biological information (Gillis et al., 2014). However, recent systematic approaches to explore PPI via AP-MS called BioPlex (Huttlin et al., 2015, 2017, 2021) and Y2H called HuRI (Rual et al., 2005; Rolland et al., 2014; Luck et al., 2020) have enabled an unbiased proteome-wide analysis of PPIs (Supplementary Table 3). BioPlex and HuRI are highly valuable interactomes for analyzing EV PPIs as they appear to have a lower localization bias compared to other networks (Luck et al., 2020). This work identified a PPI “community” of vesicular proteins, which are unbiased subnetworks significantly enriched for EV localization (Luck et al., 2020). We hypothesized that key control elements in EV biogenesis or recruitment of specific cargo would be the possible “seed protein” candidates, which exist as hub proteins that link their interacting proteins within the same EV subtypes. We predict that if these “seed proteins” exist, PPI may occur more frequently between exosomal proteins and between ectosomal proteins, than between the two of them (Figure 3A). We showed that protein interaction partners share a similar tendency to be included in sEV or lEV, showing a positive correlation between fsEV/flEV ratios of bait and prey proteins of interacting protein pairs (Figure 3B; Spearman correlation ρ = 0.1319 with *P* < 10^–42^). Moreover, a network analysis of EV proteins (proteins identified more than 50 times in EVpedia) shows that sEV proteins and lEV proteins clusters are significantly separated in space, sEV (red) and lEV (blue) proteins located in the left upper corner and right lower corner, respectively (Figure 3C). In particular, we found two examples of “seed proteins,” whose PPI subnetworks are enriched with proteins that have a higher tendency to exist in corresponding EV subtypes: SDCBP for sEVs (Figure 3D) and ATP5ME for lEVs (Figure 3E).

In our previous interactome study (Luck et al., 2020), we validated SDCBP as a hub protein whose knock-out can downregulate the abundance of its interactors in EVs, alluding to the notion that SDCBP is involved in the recruitment of other proteins into EVs. Further, there were several SDCBP-interacting proteins whose expressions were downregulated by SCDBP knockout. These downregulated proteins included CEP55, which interacts with TSG101 (Lee et al., 2008) and CALM1, and HPRT1 that were proposed as novel EV cargoes recruited by PDZ domains of SDCBP (Garrido-Urbani et al., 2016). Meanwhile, ARF proteins (especially ARF6) – which are related to ectosome biogenesis – remain enriched in EV isolates (sedimented at 100,000 × *g* without pre-clearance) after SDCBP knockout, further supporting our “seed protein” hypothesis. In conclusion, these proteomic analyses suggest that overall proteomic profiles of each EV subtype are influenced by interactions of recruiter proteins involved in biogenesis.

### The Components of Small Extracellular Vesicles and Large Extracellular Vesicles Have a Specific Biological Signature in Functional Enrichment

**FIGURE 4:**
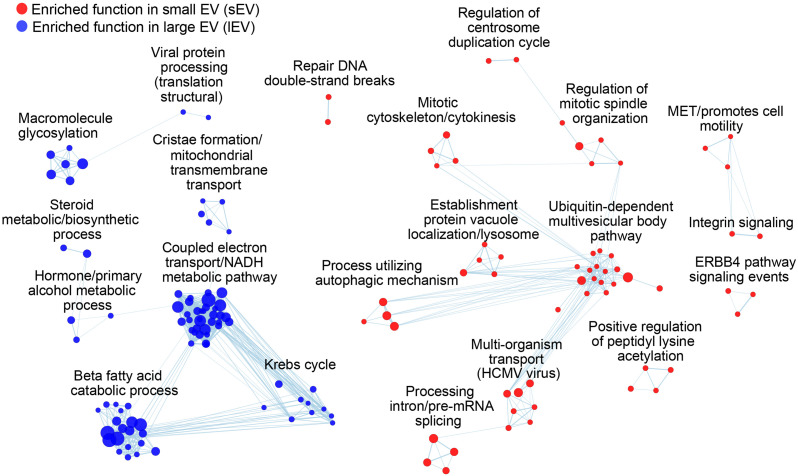
sEV and lEV proteomes are enriched with distinct biological functions and processes. Each node represents a functional annotation, where node size reflects the enrichment score. Overlapping terms are further grouped using EnrichmentMap. Functions enriched for higher identification frequencies in sEVs and lEVs are colored with red and blue, respectively.

The difference in biogenesis mechanism between exosomes and ectosomes also explains the changes in the functional enrichment in their proteomes. We sorted EV proteins according to a rank score [log2(fsEV/flEV)] that describes the tendency of vesicular proteins to be identified in sEV (positive score) or lEV (negative score) datasets. Despite the differences in EV-secreting cell types, there are specific protein cargoes enriched within each EV fraction, suggesting a functional divergence between ectosomes and exosomes (Figure 4, Supplementary Figure 2A, and Supplementary Tables 4A,B). sEV proteins showed enrichment for functions relevant to their biogenesis such as multivesicular body sorting and vacuolar transport, while also showing enrichment for cell division and extracellular signaling pathways. Proteins in lEV showed strong enrichment for mitochondrial functions (Figure 4), further supporting their previously proposed role in supplying energy to tumor microenvironments (Lazar et al., 2018). The differences in functional enrichment raise a need of studying the physiological functions of the subtypes separately, thus further highlighting a necessity for establishing robust isolation methods of EVs to fully understand their biological roles.

We also performed a functional enrichment analysis of equally abundant proteins in both lEV and sEV to reveal common biological functions shared by all EVs (Supplementary Figure 3). We filtered the dataset for proteins that showed relatively similar identifications [| log2(fsEV/flEV)| ≤ 0.1] and ranked them by their abundance in the human datasets (IC_human_). Interestingly, nucleoside metabolic processes and immune-related functional modules were highly enriched which were also independently enriched for sEV and lEV proteins along with other functions like viral protein processing (lEV) and viral transport (sEV). This finding aligns with the previously proposed dual role of EVs during viral infection in increasing host immune responses as well as the virulence of viruses (Rybak and Robatzek, 2019; Ipinmoroti and Matthews, 2020). The EV proteins were also enriched in functions such as protein folding, localization to ER, and translation (Supplementary Table 4C), suggesting that ER proteins may be loaded into EVs through interactions with common “seed proteins” related to EV secretion. Consistent with previous findings, EVs showed enrichments for exocytosis (Lin et al., 2019), secretion, and signal transduction pathways including EGF and WNT signaling pathways (Al-Nedawi et al., 2008; Li et al., 2012; Latifkar et al., 2019; Supplementary Figure 2B). Altogether, functional enrichment analysis of proteomic data can be used to investigate the underlying biological role of EVs. Furthermore, our findings suggest that we may be able to identify specific biomarkers that are upregulated in a particular disease by analyzing the differential expression in sets of functionally related proteins, rather than single target proteins.

## Discussion

Recent studies suggest that the biological properties of EVs could be exploited as a new strategy for novel biomarkers and treatment of diseases, and yet a systematic approach to validate molecular classifications of EV subtypes has been lacking. To address this need, we performed a systemic analysis of the available EV datasets to explore defining proteomic characteristics of lEVs and sEVs. Our analysis suggests that we can infer unique functions and biogenesis elements of EV subtypes from published EV proteomes, questions which were rarely explored in related studies. These results also revealed several other core features of EVs such as (i) evolutionary conservation, (ii) functional categories, and (iii) PPI networks that could be demonstrated using extensive statistical and systematic approaches.

We defined the identification frequency of proteins in sEV and lEV according to differential ultracentrifugation (DUC) speeds (Figure 1A). We used centrifugation speeds that are commonly used for separating and concentrating EVs (Kowal et al., 2016), which are referred to as sEV and lEVs instead of more specific subtypes. Although the protocols used in each proteomics study are not consistent including centrifugation steps, the ultracentrifugation speeds successfully divided datasets into sEV and lEV fractions, which had statistically significant differences in the expression of several exosome markers. There are only a handful of datasets that combined other methods along with centrifugation; therefore, it was not possible to perform meaningful analysis on this material. Instead, to allow us to use datasets to discover general characteristics of sEV and lEV proteins, we validated our classification system using the identification of the most common exosome-specific markers in the datasets (Figure 1). However, the remaining challenge of this work is that due to availability of relevant details in pertinent publications, we have not been able to include EV proteome datasets dealing exclusively with DUC or validate the status of EV quality controls mentioned in a recent publication (Théry et al., 2018).

While our method does not define EV biogenesis mechanisms, it allows a separation of EV subsets that are clearly different, a notion which has been supported by respective enrichment of known exosomal and ectosomal markers (Figures 1B,C). Despite this consistency, DUC still carries remaining limitations as an independent method to separate the EV subtypes (Witwer et al., 2013). While newer Absolute Protein Expression (APEX) Quantitative Proteomics Tool might show significant differences in the relative inclusion of markers between sEV and lEV fractions, protein concentrations are often not considered in recovered EV fractions (Braisted et al., 2008). Another impediment posed by the current purification schemes is the protein overlap between sEV and lEV fractions which is evident from our functional annotation analysis (Supplementary Figure 3). Therefore, there is a need for additional criteria to identify markers that could distinguish EV subtypes more clearly.

Little is known about the role of vesiculation in the evolutionary leap from a simple prokaryotic cell to a more complex eukaryotic cell (Embley and Martin, 2006). Indeed, mitochondria are likely evolved from engulfed prokaryotes that once lived as independent organisms (Gray et al., 1999). This hypothesis is supported by our evolutionary and functional enrichment analysis that demonstrated the inclusion of prokaryotic and mitochondrial proteins in ectosomes. Likewise, the export of cellular fragments, such as EVs, has evolved from a primordial and conserved membrane budding mechanism to a more complex pathway of exosome biogenesis (van Niel et al., 2018). We suggest that the current EV purification method, even as simple as DUC, is sufficiently robust to capture the salient differences in protein networks that separate distinct EV subtypes and their evolutionary trajectory (Figure 2). In this regard, we show that lEVs contain conserved proteins characteristic of both prokaryotes and eukaryotes, as is the process of membrane budding. In contrast, sEV proteins are characterized as being mostly eukaryotic, reflecting a more recent emergence of the endosomal vesiculation process. These correspond to our functional annotation of sEV and lEV fractions (Figure 4). Indeed, lEVs comprise proteins involved in mitochondrial function, including energy synthesis, and sEV proteins mostly function in the synthesis of intracellular building blocks and signaling pathways (Figure 4). Recent studies using TEM showed that mitochondria are transferred through microvesicles (ectosome) (Phinney et al., 2015; Liu et al., 2021). Moreover, it is also known that damaged mitochondria are transported into migrasomes to maintain the quality of the mitochondrial pool (Jiao et al., 2021). Indeed, our data demonstrate that large vesicles may contain and transfer mitochondria. Interestingly, the genes related to cellular homeostasis and stress response including control of cellular proliferation and death and control of metabolic function (protein localization, biosynthetic and processes of organic compounds, regulation of catabolic processes, and actin reorganization) are not changed between sEV and lEV (Supplementary Figure 3). This functional segregation of proteins among EV subtypes and across a large population of databases demonstrates that our method may offer a new explanation to the emergence of different classes of EVs, some of which may be relevant to the evolutionary hypothesis of an endosymbiotic relationship between cellular as well as extracellular organelles.

Due to significant differences in fsEV versus flEV, we believe that there is an apparent set of EV type-specific recruiters that divide ectosomal and exosomal components. By extensive network analysis, we showed the existence of recruiter candidates and proposed mutually exclusive EV markers, some of which were previously reported. The limitation of this work is that we have integrated EV proteomics from various cellular contexts, thereby making it difficult to apply to all EV types from all cells and organisms of origin. Since EV composition is dependent on several factors, such as the cell of origin, metabolic activity, and health and pathologic conditions, a more focused EV analysis method is needed to control for known covariates. We highlight that we can utilize distinctive proteomic characteristics of exosomes and ectosomes as an alternative to using specific molecular markers for assessing the relative abundance of the EV subtypes in the purified EV sample and validating its purity.

Due to the fundamentally divergent biogenesis of exosomes and ectosomes, it is highly probable that a set of EV subtype-specific recruiters may distinguish ectosomal and exosomal components and is subject to evolutionary changes. By extensive network analysis, we showed the existence of recruiter candidates and identified mutually exclusive EV markers (Figure 3), some of which were shown by an independent experimental study (Kugeratski et al., 2021). We demonstrate that distinctive characteristics of the sEV and lEV proteomes can offer a high granularity alternative to traditionally used singular molecular markers in assessing EV subtypes and their functions. Taken together, this method can be applied to developing new analytical approaches that, in concert with metabolomics and transcriptomics, may offer unprecedented insights into the biology, functions, and clinical applications of EVs.

## Data Availability Statement

The original contributions presented in the study are included in the article/Supplementary Material, further inquiries can be directed to the corresponding authors.

## Author Contributions

D-KK got the invitation of manuscript submission for this special issue. HL and D-KK organized the first draft of the abstract and got an acceptance from the editorial board for this special issue. HL, HY, and HK performed overall bioinformatics analyses and organized the manuscript. Y-WK, J-EK, OK, E-YL, and J-CT revised the manuscript. JR and D-KK organized the study design and finalized the manuscript. All authors contributed to the article and approved the submitted version.

## Conflict of Interest

The authors declare that the research was conducted in the absence of any commercial or financial relationships that could be construed as a potential conflict of interest.

## Publisher’s Note

All claims expressed in this article are solely those of the authors and do not necessarily represent those of their affiliated organizations, or those of the publisher, the editors and the reviewers. Any product that may be evaluated in this article, or claim that may be made by its manufacturer, is not guaranteed or endorsed by the publisher.

## Supplementary Material

Supplementary Figure 1 Comparison of the identification frequencies of conventional exosomal and ectosomal markers in **(A)** large extracellular vesicles (lEVs) and **(B)** small EVs (sEVs) datasets. n.s., not significant; ^∗^*P* < 0.05.

Supplementary Figure 2 Score plots from Gene Set Enrichment Analysis (GSEA) for **(A)** sEV (top) and lEV (bottom) proteins. **(B)** Enrichment score plots for vesicular proteins with similar identification frequencies in lEV and sEVs, using identification counts across human datasets. Upper panel, frequently identified EV proteins; lower panel, rarely identified ones.

Supplementary Figure 3 Gene Set Enrichment Analysis (GSEA) reveals functions of proteins similarly abundant in both EV subtypes.

Supplementary Table 1 An integrated EV proteomic dataset by compiling the entire list of shotgun proteomic datasets including a detailed description of the table. **(A)** Description of the proteomic datasets in EVpedia used in this study. **(B)** Definition of differential ultracentrifugation (DUC) criteria used for classifying datasets. **(C)** A list of methods used for isolation of EVs for proteomic datasets in EVpedia, and how they were classified.

Supplementary Table 2 Identification frequencies of human vesicular proteins in EVpedia. fsEV and flEV indicate the number of times the protein was identified in an sEV or lEV dataset, divided by the total number of datasets, and IC_pro_ and IC_eu_ indicate the number of times an ortholog of the protein was identified in prokaryotic or eukaryotic dataset in EVpedia.

Supplementary Table 3 List of interacting protein pairs from integrating Affinity Purification-Mass Spectrometry (AP-MS; BioPlex) and Yeast Two-Hybrid (Y2H; HuRI) datasets, including table description and details of the table.

Supplementary Table 4 List of functional annotations enriched in proteins more frequently identified in **(A)** sEV and **(B)** lEV datasets. **(C)** Functional enrichment for proteins similarly identified in sEV and lEV was analyzed using their identification count across human datasets in EVpedia. The table contains a detailed description of the table. ES, enrichment score; NES, normalized enrichment score; NOM, nominal; FDR, false discovery rate; FWER, family-wise error rate.
