# Supplementary figures and images for: Extracellular Vesicle Proteomes Shed Light on the Evolutionary, Interactive, and Functional Divergence of Their Biogenesis Mechanisms
Source: Front Cell Dev Biol. 2021 Oct 1;9:734950. doi: 10.3389/fcell.2021.734950 (PMC8517337; doi:10.3389/fcell.2021.734950)

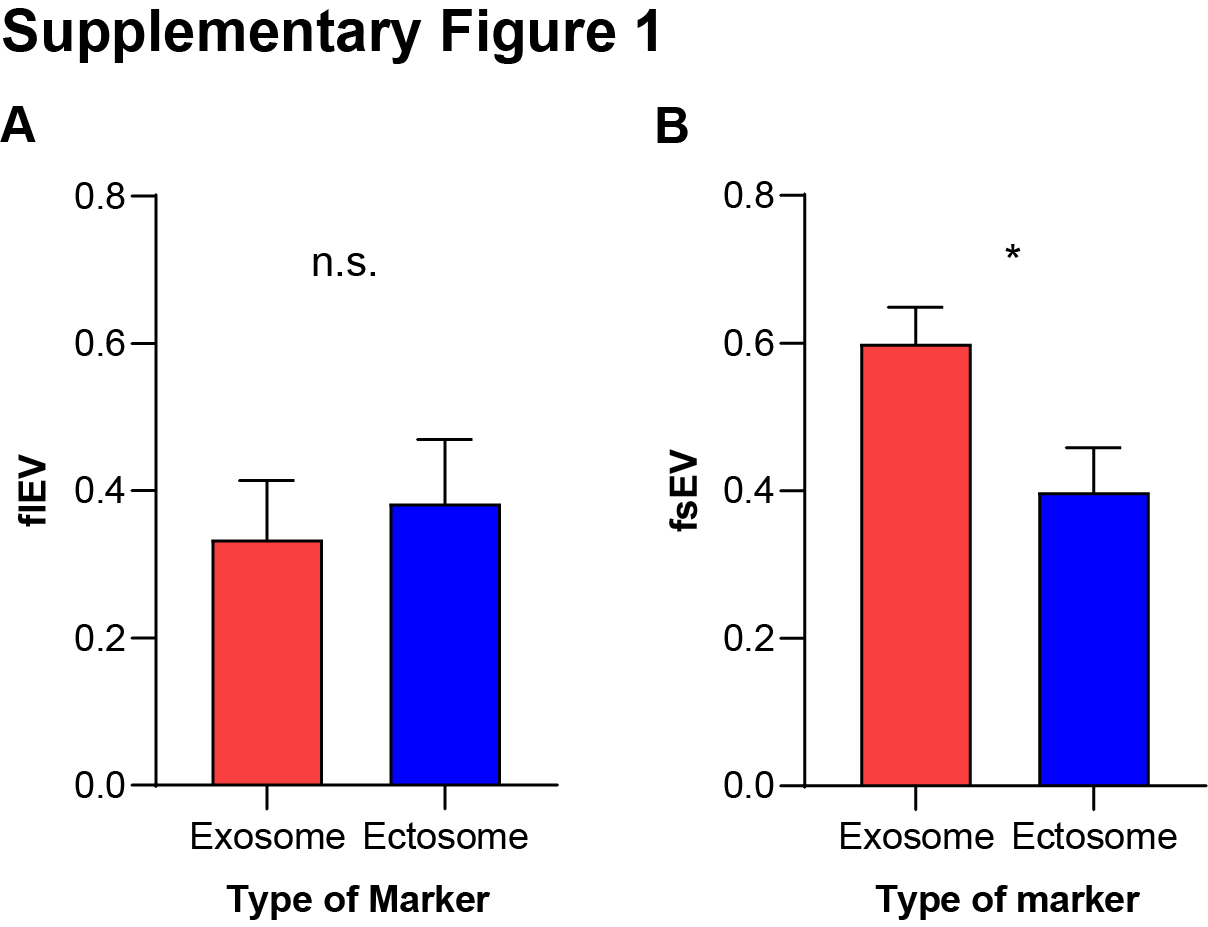

Supplement: Supplementary Figure 1 — Comparison of the identification frequencies of conventional exosomal and ectosomal markers in (A) large extracellular vesicles (lEVs) and (B) small EVs (sEVs) datasets. n.s., not significant; ∗P < 0.05. [file Image_1.JPEG]

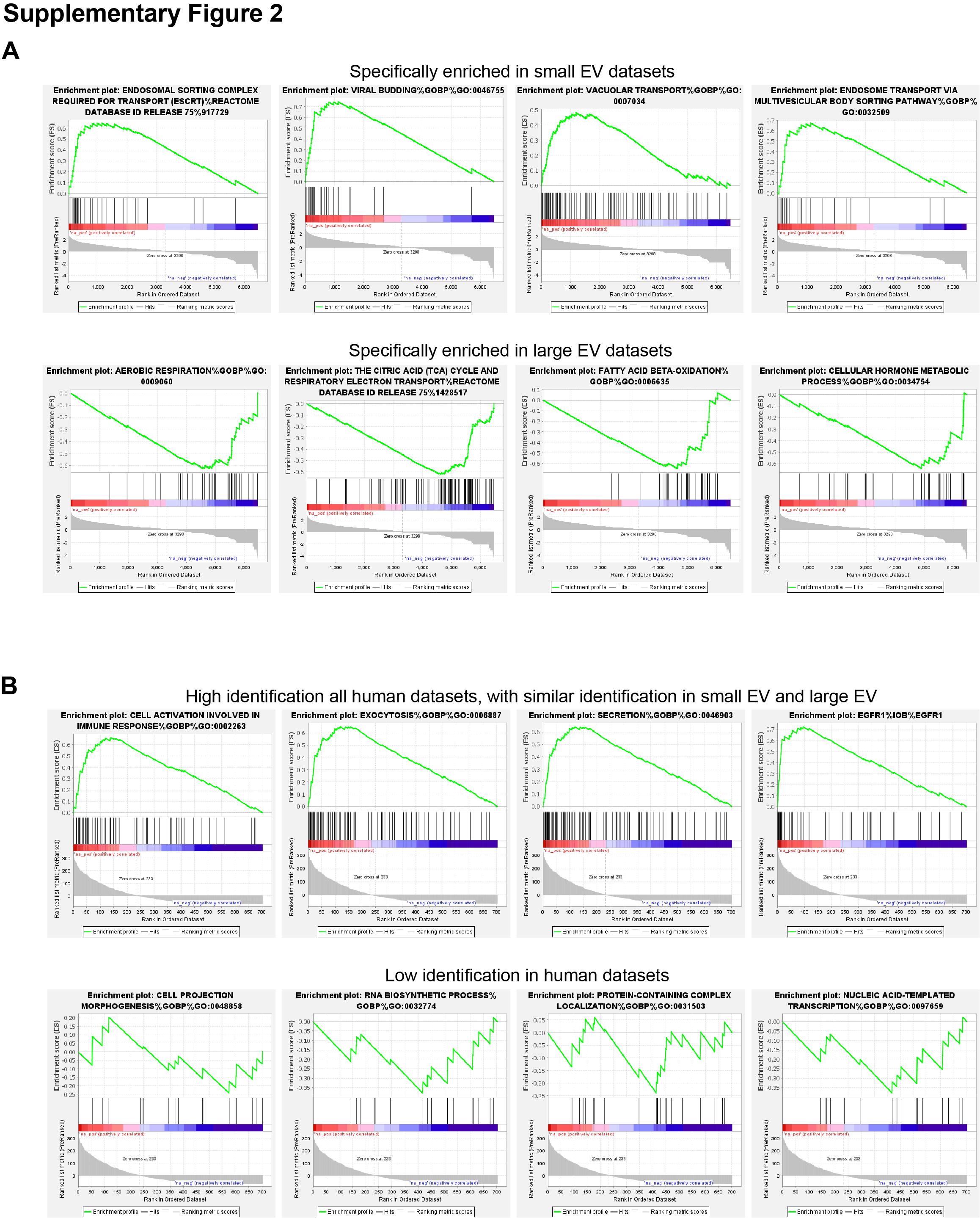

Supplement: Supplementary Figure 2 — Score plots from Gene Set Enrichment Analysis (GSEA) for (A) sEV (top) and lEV (bottom) proteins. (B) Enrichment score plots for vesicular proteins with similar identification frequencies in lEV and sEVs, using identification counts across human datasets. Upper panel, frequently identified EV proteins; lower panel, rarely identified ones. [file Image_2.JPEG]

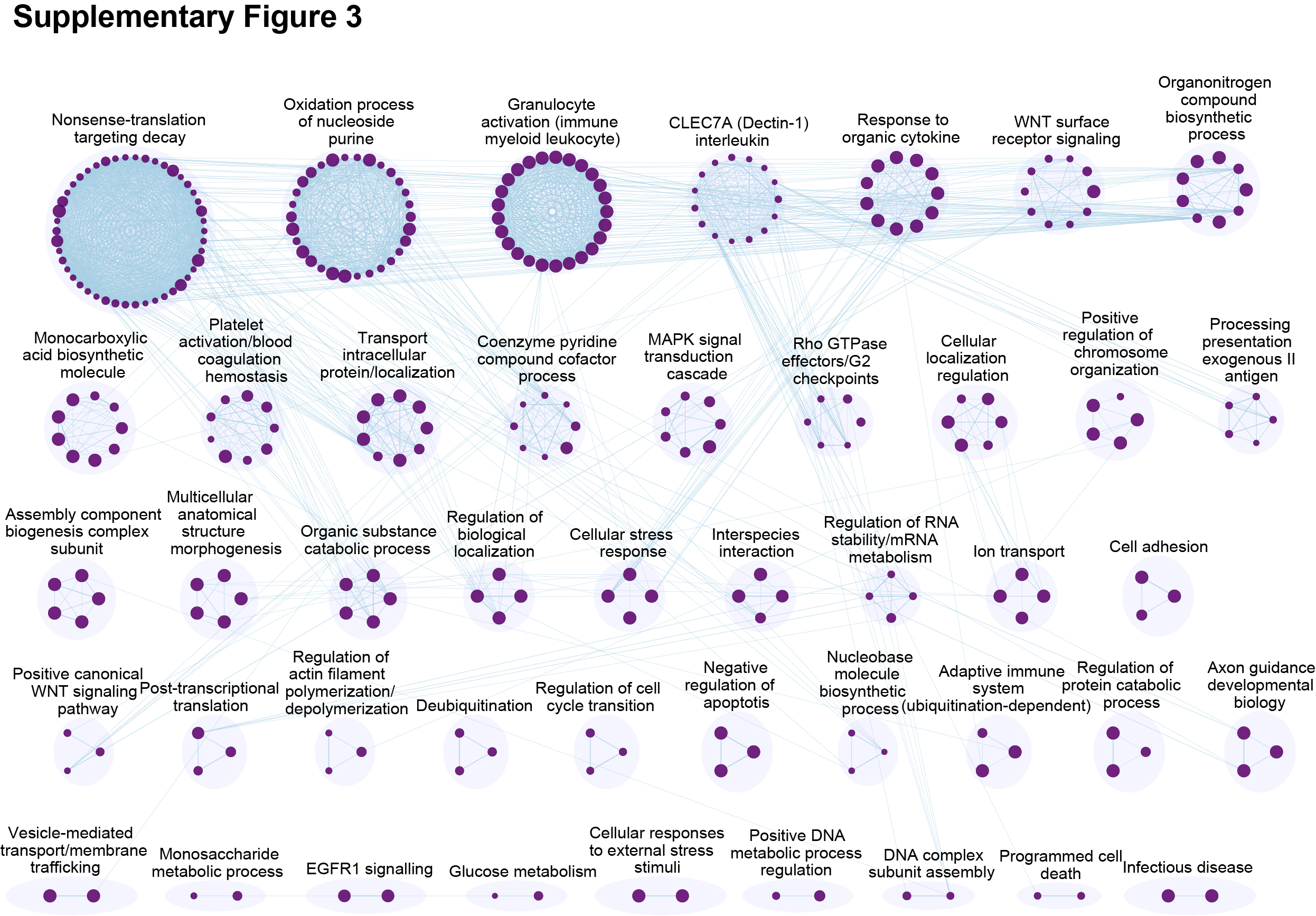

Supplement: Supplementary Figure 3 — Gene Set Enrichment Analysis (GSEA) reveals functions of proteins similarly abundant in both EV subtypes. [file Image_3.JPEG]
